# Thickness and width of the menisci of adult knee joint: a descriptive cross-sectional observational study in cadavers

**DOI:** 10.12688/f1000research.128395.2

**Published:** 2024-04-08

**Authors:** B.V. Murlimanju, S. Vikram, Vanishri Nayak, Nandini Bhat, Mangala M. Pai, Rajanigandha Vadgaonkar, Latha V. Prabhu, Sunil Nayak

**Affiliations:** 1 Department of Anatomy, Kasturba Medical College, Mangalore, Manipal Academy of Higher Education, Manipal, Karnataka, India; 2 Department of Anatomy, College of Medicine, King Khalid University, Abha, Saudi Arabia, 61421, Saudi Arabia; 3 Department of Anatomy, Kasturba Medical College, Manipal, Manipal Academy of Higher Education, Manipal, Karnataka, 576104, India; 4 Department of Oral and Maxillofacial Surgery, Manipal College of Dental Sciences, Manipal, Manipal Academy of Higher Education, Manipal, Karnataka, 576104, India

**Keywords:** allografts; arthroscopic meniscectomy; tibial meniscus; transplants

## Abstract

**Background:**

The goal was to determine the thickness and width of the knee joint meniscus at their different regions. The objective was to compare the dimensions at these regions and over the right- and left-sided specimens.

**Methods:**

The present study included 50 adult cadaveric knee joints, and 100 menisci (50 medial menisci and 50 lateral menisci) were studied. The meniscus was distributed into anterior, middle and posterior parts. Thickness and width at the mid-point of these three parts were determined by using the Vernier caliper.

**Results:**

The breadth of the medial meniscus was 8.38 ± 1.64 mm, 7.68 ± 1.92 mm and 13.93 ± 2.69 mm at the anterior, middle and posterior one-third regions. Same measurements for the lateral menisci at these regions were 9.84 ± 1.78 mm, 8.82 ± 2.01 mm and 10.18 ± 2.23 mm, respectively. The thickness of the medial meniscus was 4.49 ± 0.78 mm, 4.07 ± 0.81 mm and 4.79 ± 0.93 mm at these regions. The lateral meniscus thickness was 3.82 ± 0.69 mm, 4.43 ± 0.98 mm and 4.36 ± 0.8 mm, respectively.

**Conclusion:**

It is believed that this data is enlightening to the arthroscopic surgeon during the meniscus transplantation either by using synthetic material or allograft as the proper sizing of the meniscus is important to prevent complications due to inaccurate sizing.

## Introduction

The investigation of intracapsular ligaments of knee joint is important in the clinical setup to make accurate diagnosis and surgical procedures of the knee joint. ^1^ There has been a substantial rise in the information about the arthroscopic management of the meniscal injuries. ^2^ The menisci can be easily injured and it is hard to repair them. ^3^ However, due to advanced technologies, the torn menisci can be repaired by arthroscopic surgery and the best modality of treatment available is the meniscal allograft transplantation, especially for the young symptomatic patients. ^4^ At present, arthroscopy can involve meniscal transplantation, scaffold implantation and insertion of healing substances at the torn area of the meniscus. ^5^ Meniscectomy can be performed arthroscopically and it is followed by synthetic or allograft meniscal transplantation as this prevents friction between the tibia and the femur. ^6^ It was opined that successful meniscus transplant surgery relies on the accuracy in the dimensions matching between the recipient and donor meniscus. ^7^ Precision of the measurements between the allograft and the recipient knee is important for successful function of the meniscus after the transplantation surgery. If the graft size is small, forces acting on the meniscus are increased, which may lead to misbalancing of the femoral condyles and compartment overload. ^8^ If the allograft is larger, there will be no load bearing and the forces will be increased over the articular cartilages of the knee joint, which causes degenerative changes in the knee joint. ^7^ It was reported that only 5-10% of the mismatch of the graft size is accepted. ^9^

The morphometric data of menisci will also assist the biomechanical research and hypothesis, which explores the functional relationships of the femoro-tibial articulation. ^10^ This has implications for tissue engineering of the meniscus and offering the normal joint function. There are clinical studies, which suggest that radiological measurements of the menisci need to be compared with the menisci from cadaveric specimens to confirm and make the radiological method as the gold standard. ^7^ The morphological information about the sizes of the meniscus can benefit in differentiating the normal meniscus from the discoid and tiny meniscus. ^11^ The bucket handle meniscal tear decreases the width, so it is important to differentiate the torn meniscus from the normal meniscus in the radio diagnosis. It was reported that, alterations in the width and thickness of meniscus can give clue to the type of its injury and impact. ^12^ Smillie ^13^ described that the thickness and width of the menisci are important to understand the possibility, location and type of meniscal injury. The morphological variability between the meniscus of medial and lateral compartment is important to understand the meniscal damage. ^13^

The operating surgeons should ask the tissue providing banks for the allograft, which matches with the dimension of the torn meniscus. ^7^ In this context, the normative data about the breadth and thickness of the menisci may help in choosing the accurate meniscal graft. Due to these clinical implications, the objective of the present research was to measure the breadth and depth of the meniscus in corpses and provide normative data of this sample population. The objective was to correlate the dimensions over the three different regions of meniscus and over the right- and left-sided specimens.

## Methods

This study was a descriptive cross-sectional observational research, performed at the department of anatomy of Kasturba Medical College, Manipal Academy of Higher Education, Manipal, India. All the strobe guidelines for the cross-sectional study are met and the details are available at https://figshare.com/articles/dataset/Strobe_Checklist/21605694. The study participants were formalin fixed human adult cadaveric knee joints of unknown age and gender. Only embalmed undissected lower extremity specimens were included in this study. The specimens, which exhibited the visible pathological changes and congenital anomalies were excluded. The study duration was one year from 23.08.2021 to 23.08.2022. The sample size was 50 adult cadaveric knee joints; amongst them 25 were right- sided and 25 left-sided specimens. The sample size of this present study was calculated by the formula 'n = Zα ^2^ x σ ^2^/d ^2^', where Z _α_= 1.96 at 95% confidence level, σ = standard deviation, d= 1% absolute precision with 95% confidence level and 80 % power with 1% absolute precision, the sample size comes to be 100. The reference article used to calculate the sample size was El-Aasar *et al*. ^14^

In total, 100 menisci were studied (50 medial menisci and 50 lateral menisci). The gender of the specimens were not taken into consideration. The cadaveric lower limbs were frozen, which were initially embalmed with 10% formalin. After dissecting the skin, fascia and muscles, a longitudinal cut was given on the medial and lateral sideways of the knee joint to open the capsule. The ligamentum patellae, tibial and fibular collateral ligaments were cut by giving a transverse incision. The knee capsule and cruciate ligaments were cut for better access to the menisci, while the femoral and tibial condyles were separated from the soft tissues, which exposed the whole menisci. The knee joints which showed pathological changes and were found damaged were excluded from the present study. The knee joints exhibiting congenital anomalies and tears of the menisci were also disqualified from the present research. This present research followed the guidelines of the international ethical standards as per Padulo *et al*. ^15^

**Figure 1.**
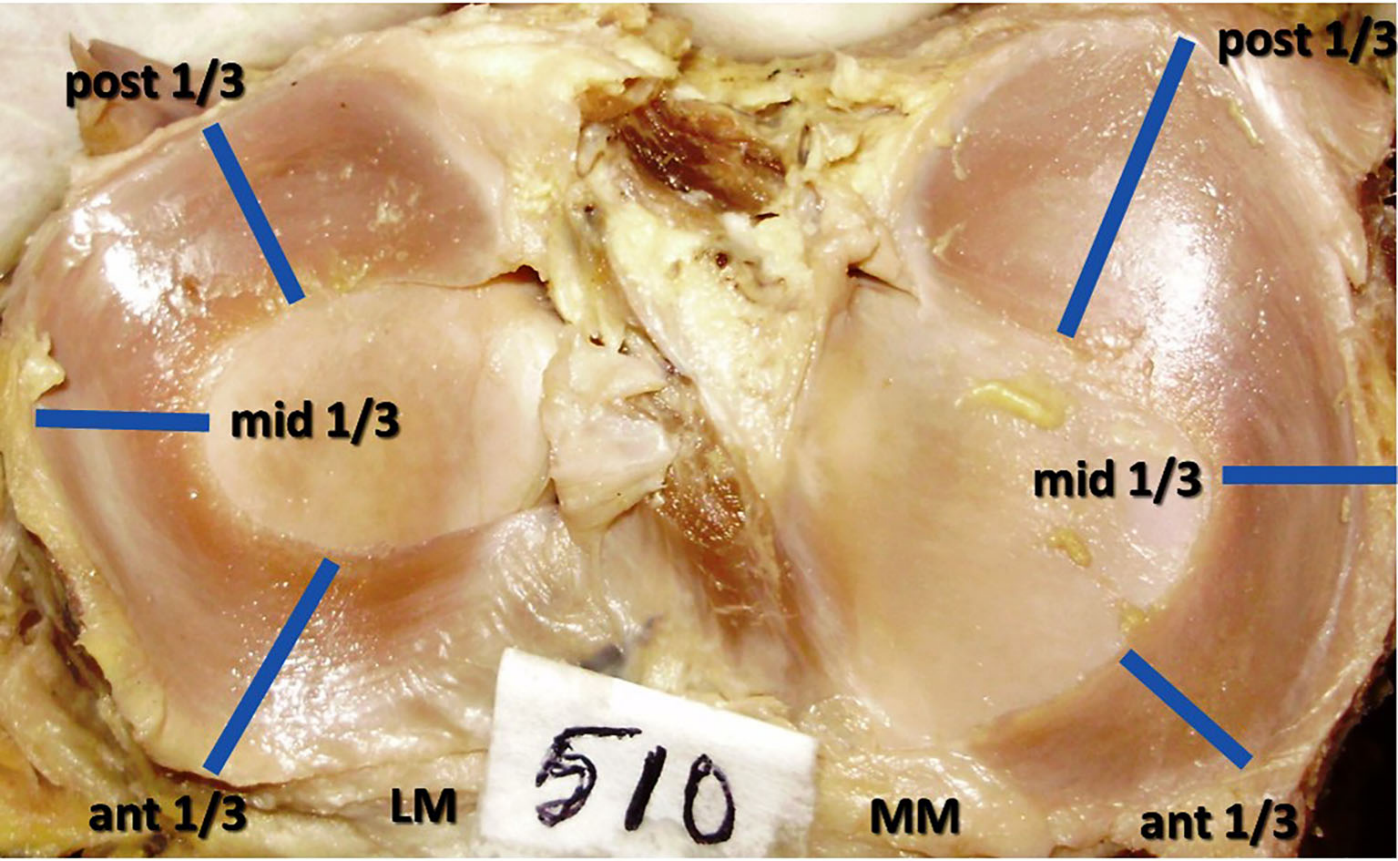
Anterior, middle, and posterior thirds, the midpoints of these 3 regions where the measurements of width and thickness of the menisci were performed and analyzed.

The peripheral length of the menisci was measured by using a thread and the measuring scale. The total peripheral length was divided into three components, anterior 1/3, middle 1/3 and posterior 1/3 regions ( Figure 1). The width and thickness of these three regions were measured at the midpoint of each regions. This is as per the previous publication by Almeida *et al.* ^12^ This was approximately corresponding to 1 o'clock, 3 o'clock and 5 o'clock positions for the medial meniscus and 7 o'clock, 9 o'clock and 11 o'clock positions for the lateral meniscus of the right knee joints. For the left knee joints, it was 7 o'clock, 9 o'clock and 11 o'clock positions for the medial meniscus and 1 o'clock, 3 o'clock and 5 o'clock positions for the lateral meniscus.

The measurements were performed with the vernier caliper of 0.02 mm accuracy. At the mid-point of each 1/3, the caliper was positioned between the outer and inner borders of the menisci to measure the width. The thickness was measured at the same points by placing the caliper between the upper and lower surfaces of the menisci at the outer edge only. All the dimensions were completed by the same researcher to avoid the inter observer variation and three readings were taken for each measurement to prevent the intra-observer error.

The data were tabulated in the microsoft excel (RRID:SCR_016137) document and presented as mean ± standard deviation. The online SPSS (RRID:SCR_002865) program (version number 25) was utilized for the analysis. The calculation of values and statistical analysis between the right versus left sides and medial versus lateral tibial partitions were done by using the paired samples ‘t’ test and repeated measures ANOVA (online software SPSS (RRID:SCR_002865), version number 25, https://www.ibm.com/products/spss-statistics-gradpack). The ‘p’ value less than 0.05 was considered as significant (α = 0.05). The protocol of this research is available online at https://www.protocols.io/view/thickness-and-width-of-the-menisci-of-adult-knee-j-cjh2uj8e.

## Results

The thickness and width of the medial meniscus at the 3 different regions are tabulated and compared among the right and left sides ( Table 1). Table 2 shows the morphometric data and comparison of lateral meniscus among the left and right knee joints. The present study observed that there was no significance in this comparison for the thickness and width between the corresponding right and left knee menisci, in both medial and lateral compartments (p>0.05).

**Table 1.** Showing the comparison of the morphometric data of medial meniscus on right and left knee joints (n=23).

parameter	region	right side	left side
	ant 1/3	8.51 ± 1.65	8.09 ± 1.69
width	mid 1/3	7.77 ± 2.24	7.46 ± 0.92
	post 1/3	14.27 ± 2.61	12.9 ± 2.99
	ant 1/3	4.43 ± 0.74	4.62 ± 0.92
thickness	mid 1/3	3.99 ± 0.89	4.29 ± 0.55
	post 1/3	4.84 ± 1.05	4.61 ± 0.31

values are in mms; mean ± standard deviation; statistical significance (paired t-test).

**Table 2.** Showing the comparison of the morphometric data of lateral meniscus on right and left knee joints (n=23).

parameter	region	right side	left side
	ant 1/3	9.63 ± 1.88	10.33 ± 1.55
width	mid 1/3	8.69 ± 2.22	9.08 ± 1.5
	post 1/3	10.31 ± 2.35	9.79 ± 2.02
	ant 1/3	3.64 ± 0.66	4.04 ± 0.77
thickness	mid 1/3	4.59 ± 0.93	3.99 ± 1.08
	post 1/3	4.39 ± 0.75	3.94 ± 0.9

values are in mms; mean ± standard deviation; statistical significance (paired t-test).

The medial and lateral menisci were compared to each other irrespective of their side and the comparison is given in Table 3. Related to the peripheral circumference thickness, the anterior one-third region of medial meniscus is thicker than the lateral (p<0.05). But in the middle and posterior third regions ( Table 3), medial and lateral menisci are of relatively same thickness (p>0.05). In the anterior and middle third regions, the lateral meniscus has additional width than the medial (p<0.05). But in the posterior one-third region, medial meniscus was wider (p<0.05), than the lateral meniscus ( Table 3).

**Table 3.** Showing the comparison of the morphometric data of menisci (n=46) on medial and lateral compartments of the knee joint.

parameter	region	medial meniscus	lateral meniscus
	ant 1/3 *	8.38 ± 1.64	9.84 ± 1.78
width	mid 1/3 *	7.68 ± 1.92	8.82 ± 2.01 *
	post 1/3 *	13.93 ± 2.69 *	10.18 ± 2.23
	ant 1/3 *	4.49 ± 0.78	3.82 ± 0.69
thickness	mid 1/3	4.07 ± 0.81	4.43 ± 0.98
	post 1/3	4.79 ± 0.93 *	4.36 ± 0.8

values are in mms; mean ± standard deviation; statistical significance (repeated measures ANOVA).

* p<0.05.

**Figure 2.**
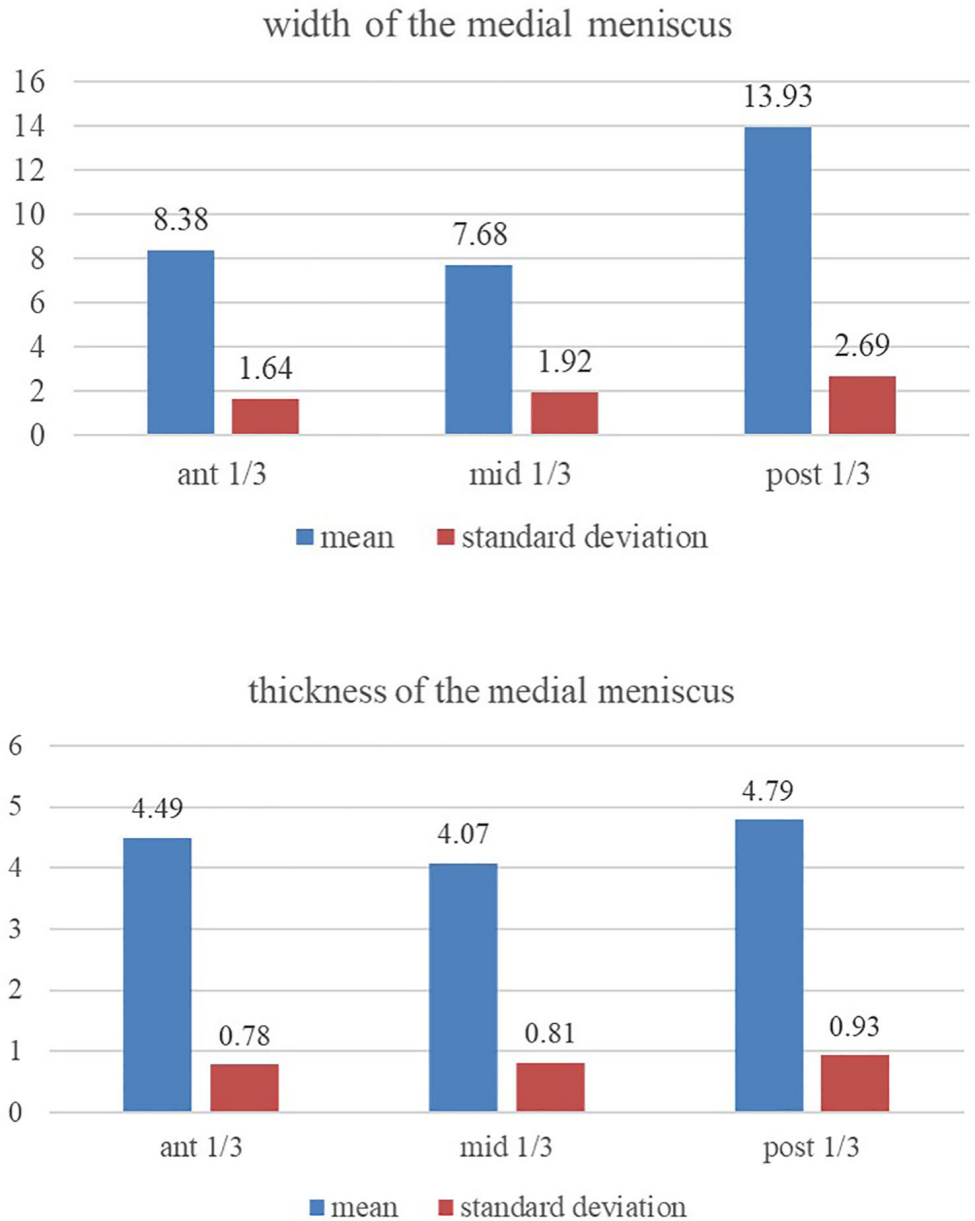
Showing the comparison of the width and thickness of the medial meniscus at its anterior, middle and posterior one-third regions (statistical test-repeated measures ANOVA).

The posterior one-third region of the medial meniscus is thicker ( Figure 2) than its anterior and middle one-thirds (p<0.05). The middle and anterior one-third regions are of the same relative thickness (p>0.05). The posterior one-third region of the medial meniscus has greater width ( Figure 2) than its own anterior and middle one-thirds (p<0.05). The anterior and middle one-third regions were of the same relative width (p>0.05).

**Figure 3.**
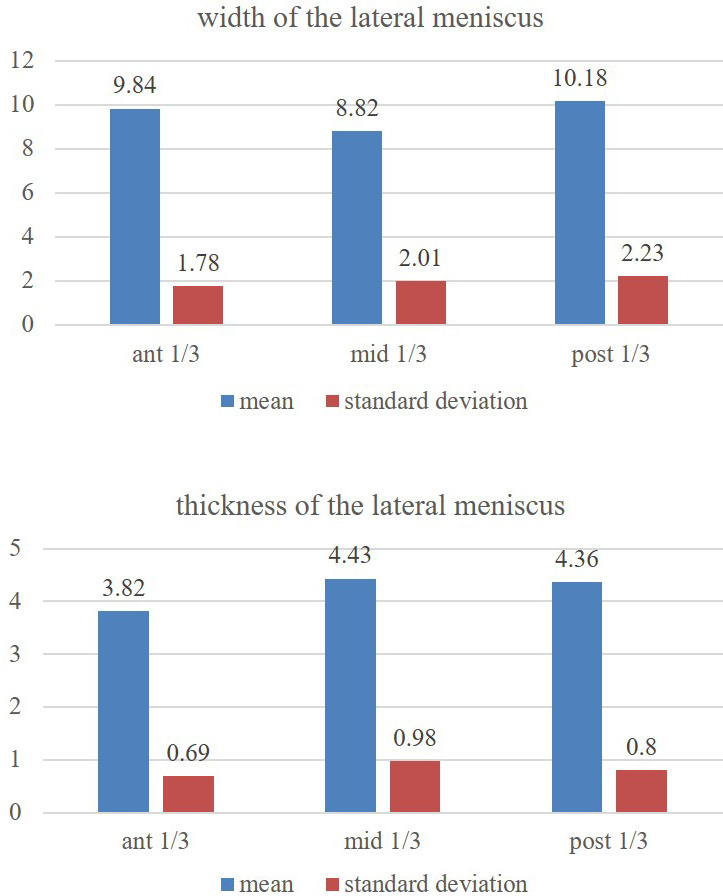
Showing the comparison of the width and thickness of the lateral meniscus at its anterior, middle and posterior 1/3 one-third regions (statistical test-repeated measures ANOVA).

The statistics did not reveal the differences (p>0.05) amongst the thicknesses of anterior, middle and posterior parts of lateral meniscus ( Figure 3). However, the ventral and dorsal one-third regions of lateral meniscus ( Figure 3) are of the same relative width (p>0.05) and they are greater than the middle one-third region (p<0.05).

After taking the average from all the three regions, the breadth of medial and lateral meniscus was 9.69 ± 1.89 mm and 9.61 ± 1.77 mm, respectively. The average thickness of the medial and lateral meniscus was 4.39 ± 0.67 mm and 4.17 ± 0.58 mm, correspondingly. With respect to the average dimensions of width and thickness, the statistical significance was not observed (p>0.05) between the medial and lateral meniscus.

## Discussion

Meniscal damages are among the most frequent harms in the sports medicine, which can cause pain, functional disability and swelling around the knee joint. ^16^ The meniscal tears occur due to the bending forces and rotational knee injuries. They can also occur due to degeneration or spontaneously due to the progressive structural failure, which is known as the meniscal fatigue injury. ^17^ Knee arthroscopy is the gold standard investigation, which can simultaneously treat the meniscus disorders. ^3^ The graft sizing, precise positioning and fixation are the three prerequisites for successful allograft meniscal transplantation. ^18^ In allograft meniscal transplantation, which is performed for the torn menisci, inaccurate size of the graft can lead to its failure and cause complications like knee joint degeneration. Successful arthroscopic meniscal transplantation and regeneration depend on the accuracy in the shape and size of the allograft in comparison to the torn meniscus. This requires the morphometric data of the menisci as the mismatching leads to surgical failure and degenerative changes in the knee joint. ^9^^,^^19^^–^^20^ The normative data of dimensions of the meniscus are essential while planning the surgical interventions of the knee joint. This can help in differentiating the discoid meniscus from a normal one. The discoid meniscus is an anomaly and develops due to the variability in the morphogenesis. ^21^ It was reported that, variability in the thickness and width of menisci can lead to the meniscal injury. ^12^ Meniscectomy has long-term complications like joint degeneration, and it is advisable to do conservative surgery by repairing the torn meniscus, but for this, grafting is required. Accurate size of the meniscal allograft is required for the success of the arthroscopic surgery in terms of the joint function and healing. The correct sizing of the graft requires detailed knowledge about the dimensions of the menisci.

In this perspective, the present cadaveric study has provided normative data about the thickness and width of the meniscus in this sample population of Indians. The complete set of data of the present study was compared with the previous studies from other populations and is represented in Table 4. The observations of the present study agree with the findings of Didio, ^22^ Braz and Silva ^23^ that the posterior region was the broadest among all the three regions in the medial meniscus. In our previous study from fetal knee joints also, it was found that the posterior part was the wider of the medial meniscus. ^24^ In this research from Indian samples, significant difference was not obtained among the anterior and middle third widths. This was similar to our finding in the previous study in fetal medial meniscus. ^24^ However, Braz and Silva ^23^ stated that the mid body of the medial meniscus has greater width than the anterior one-third.

**Table 4.** Comparison of the data of the present study with the previous reports from other population.

authors	population	width		thickness	
		medial meniscus	lateral meniscus	medial meniscus	lateral meniscus
present study	India	9.69 ± 1.89 mm	9.61 ± 1.77 mm	4.39 ± 0.67 mm	4.17 ± 0.58 mm
Bloecker *et al*. ^10^	Austria	9.9 ± 1 mm	10.1 ± 1.2 mm	2.82 ± 0.3 mm	2.67 ± 0.3 mm
Braz and Silva ^23^	Brazil	10.65 mm	11.38 mm	5.88 mm	5.46 mm
Testut and Latarjet ^25^	Spain	10.84 mm	10.72 mm	6 mm	8 mm

Almeida *et al*., ^12^ Braz and Silva ^23^ reported no statistical significance in the comparison of the width between the three regions of the lateral meniscus. We observed that the anterior and posterior one-third regions of lateral meniscus were of the same relative width. However, these two regions were wider than the middle one-third region. In the fetal lateral meniscus, it was a different observation, where the middle region was the widest. ^24^

*In vivo* radiological research by Bloecker *et al*. ^10^ revealed that medial meniscus has greater thickness than the lateral. However, there was no significance in the differences with respect to the breadth. Testut and Latarjet, ^25^ and Didio ^22^ reported that, meniscus of the medial side is wider than the lateral, when the average measurement was taken. Miller ^26^ also had the same observations in his study. However, the present study does not agree with these opinions as we didn’t find differences between the medial and lateral compartments, when the average was taken from all the three regions.

Figueroa *et al*. ^27^ observed that, the anterior region of lateral meniscus is wider than the medial meniscus. In this study, dissimilarity was not observed between the medial and lateral meniscus, average widths. Dhananjaya *et al*. ^28^ reported the *in vivo* measurements of the menisci in south Indian population. In their study, it was obvious that the lateral meniscus had higher breadths at the anterior and middle regions than its counterpart. They also detected that the posterior region of the medial meniscus had higher width than the lateral meniscus in the same region. They noted that the medial and lateral menisci were wider at their posterior region than the anterior region. The middle one-third region was relatively lesser in width.

However, the present study agrees with the finding of Braz and Silva ^23^ that the anterior region of the medial meniscus has greater thickness than that of the same part of the lateral meniscus. Our study agrees with the Bloecker *et al*. ^10^ that the thickness of the middle part of the medial and lateral menisci was the same. We further observed that the thickness of the dorsal part of the medial and lateral menisci was the same. This is not as per the findings of Bloecker *et al*., ^10^ where posterior region of medial meniscus was thicker than its counterpart. The study of fetal knee joints did not reveal statistically significant variance with respect to the thickness of the menisci of the medial and lateral compartments. ^24^ However, the lateral meniscus had greater width than its counterpart in the medial compartment in the fetal tibia.

Rico and Ayala ^29^ opined that the middle part of the medial menisci is prone to tears (51%), which is followed by the posterior one-third (39%). This was supported by the anatomical studies, and it was hypothesized that thicker region of the meniscus is more prone for tears. Braz and Silva ^23^ stated that, middle region of the medial meniscus was thicker, which is trailed by the posterior and anterior regions. Almeida *et al*. ^12^ published that the anterior end of medial meniscus was thicker, followed by the posterior and middle regions. The present study is not in agreement with this observation that, we found posterior one-third region of the medial meniscus is thicker than its anterior and middle one-thirds. This supports the clinical findings of Miller ^26^ that the longitudinal tears of the menisci were more common at the posterior region. The meniscal thickest region is prone to the friction between the condyles of femur and tibia. In the study by using the fetal knee joints, the anterior region of medial meniscus was thicker in comparison to the posterior and middle regions. ^24^

Miller ^26^ reported that, middle part of the lateral meniscus is prone to radial tears. Braz and Silva ^23^ stated that, middle region of lateral meniscus was thicker, which is trailed by the posterior and anterior regions. Almeida *et al*. ^12^ also described the same observations as Braz and Silva. ^23^ The present study observed a different finding that the statistically significant difference was not observed between the thicknesses of the three regions of the lateral menisci. This is similar to the observations of our previous study from fetal specimens. ^24^

In this anatomical investigation, breadth and thickness measured at the three locations of lateral and medial menisci over the right and left knee joints did not show statistical significance. This confirms the opinion of Dargel *et al*. ^30^ that, there is a similarity in the dimensions of the right and left knees, even though they are asymmetrical. Hence, the contralateral radiological measurement of the corresponding meniscus may be considered while choosing the meniscus allograft. Prodromos *et al*. ^31^ suggested that, during the transplantation surgery, the contralateral meniscal dimensions measured by the magnetic resonance imaging can help in choosing the graft. The present study supports this opinion as there was no statistical significance observed between the comparison of these parameters.

It was reported that, there were no significant differences observed between the anatomic and radiographic methods, with respect to the measurements of the meniscus. ^31^ The best measurement method is direct measurement of the specimen during allograft harvesting. ^32^ There will be corrections for the magnification in the radiological softwares, which are used for the measurements. This will offer the approximate size of the meniscus.

## Implications and Limitations

### Implications

The meniscal injuries are commonly observed as sports injuries like in soccer, cricket and baseball players. The arthroscopy is playing a paramount part in the treatment of these traumatic lesions. It is believed that the morphometric data of this research will be of help to the arthroscopic surgeons during the surgical repair of the torn meniscus and allograft meniscal transplantation. The data procured here can be of help in the manufacture of the artificial implants.

### Limitations

The limitation of the present study includes the smaller sample size, with respect to this group of Indian sample population. The age wise and gender-based comparison was also not performed in this study. The right and left embalmed knee joints were not from the same individual in this cadaveric research. For the paired test, the best would be to have the right and left samples from the same individual. This study utilized embalmed cadaveric specimens, in which formalin may alter the dimensions of the menisci and during the disarticulation of the femur and tibia, the anatomy of the menisci might have been disturbed, which may alter the width and thickness of the menisci.

## Conclusion

Best meniscal arthroscopic transplant surgeries depend on the correct sizing of the allografts or synthetic material. In this context, the present study has provided morphometric data of the menisci of the lateral and medial compartments in detail with respect to their width and thickness at the anterior, middle and posterior regions. We believe that these data are of help to the arthroscopic surgeon during the comparison of the radiological measurement with the cadaveric measurement and assessing the graft selection.

## Ethical Approval

The ethics committee of our institution approved this research (Ethical Committee Name - Kasturba Medical College and Kasturba Hospital Institutional Ethics Committee, Approval Number - IEC: 284/2021, dated 31.12.2021). Since this is a study from the cadaveric samples, the consent from the participants is not applicable. This was waived by our institutional ethics committee.

## Consent

Since this is a cross-sectional study from the donated cadavers and did not reveal the identity of the body donor, the written informed consent was not taken from the body donor’s family for the use and publication of this research.

## Data Availability

Figshare: Medline database search strategy for ‘Thickness and width of the menisci of adult knee joint, a cadaveric study’ https://doi.org/10.6084/m9.figshare.21383763.v1. ^33^ The project contains the following underlying data:
- (file name - master chart 6.xls). Supplementary figure is available at https://www.protocols.io/view/thickness-and-width-of-the-menisci-of-adult-knee-j-cjh2uj8e. Figshare: STROBE checklist for ‘[Thickness and width of the menisci of adult knee joint: a descriptive cross-sectional observational study in cadavers]’. https://figshare.com/articles/dataset/Strobe_Checklist/21605694 Data are available under the terms of the Creative Commons Zero “No rights reserved” data waiver (CC0 1.0 Public domain dedication).
