# Supplementary material for: Thickness and width of the menisci of adult knee joint: a descriptive cross-sectional observational study in cadavers
Source: F1000Res. 2024 Apr 8;11:1573. Originally published 2022 Dec 23. [Version 2] doi: 10.12688/f1000research.128395.2 (PMC11079982; doi:10.12688/f1000research.128395.2)
Supplement: Peer review history

# Reviewer report: Reviewer response for version 2

**DOI:** 10.5256/f1000research.164519.r264024

**Published:** 2024-04-11

**Authors:** C.S Ramesh Babu

**Affiliations:** 1 Muzaffarnagar Medical College, Muzaffarnagar, Uttar Pradesh, India

Authors have fully addressed the issues raised in the earlier review and added required changes satisfactorily.

Is the work clearly and accurately presented and does it cite the current literature?

Yes

If applicable, is the statistical analysis and its interpretation appropriate?

Yes

Are all the source data underlying the results available to ensure full reproducibility?

Yes

Is the study design appropriate and is the work technically sound?

Partly

Are the conclusions drawn adequately supported by the results?

Yes

Are sufficient details of methods and analysis provided to allow replication by others?

Partly

Reviewer Expertise:

Radiological Anatomy and Clinical Anatomy

I confirm that I have read this submission and believe that I have an appropriate level of expertise to confirm that it is of an acceptable scientific standard.

# Reviewer report: Reviewer response for version 1

**DOI:** 10.5256/f1000research.140980.r243696

**Published:** 2024-02-28

**Authors:** C.S Ramesh Babu

**Affiliations:** 1 Muzaffarnagar Medical College, Muzaffarnagar, Uttar Pradesh, India

The article adds morphometric data useful for selecting appropriate meniscal allograft transplant. Measurements of width and thickness of the menisci were done dividing the menisci into anterior, middle and posterior parts but authors failed to explain the exact method used nor provided any reference where such a method was described. Authors have also mentioned "this was approximately corresponding to 1 o'clock, 3 o'clock and 5 o'clock positions" but failed to add for which meniscus - lateral or medial / right side or left side. It is suggested that the exact method adopted by the authors to divide the menisci into three parts be added in the update. Coronal MRI images of the menisci appear triangular with a thick peripheral border and a thin inner border. How can the measurements made at MRI examination of normal knee joint of the recipient be used for selection of a cadaveric meniscal allograft?

Is the work clearly and accurately presented and does it cite the current literature?

Yes

If applicable, is the statistical analysis and its interpretation appropriate?

Yes

Are all the source data underlying the results available to ensure full reproducibility?

Yes

Is the study design appropriate and is the work technically sound?

Partly

Are the conclusions drawn adequately supported by the results?

Yes

Are sufficient details of methods and analysis provided to allow replication by others?

Partly

Reviewer Expertise:

Radiological Anatomy and Clinical Anatomy

I confirm that I have read this submission and believe that I have an appropriate level of expertise to confirm that it is of an acceptable scientific standard, however I have significant reservations, as outlined above.

# Reviewer report: Reviewer response for version 1

**DOI:** 10.5256/f1000research.140980.r166822

**Published:** 2023-07-14

**Authors:** Narendra Pamidi

**Affiliations:** 1 Jeffrey Cheah School of Medicine and Health Sciences, Monash University Malaysia, Kuala Lumpur, Malaysia

This anthropometric study involves measuring the dimension of the menisci of the human knee joint. The sample size (50 cadavers) was good enough to derive statistically significant data to be useful for clinical evaluation. The data on measuring the menisci can help diagnose meniscal tears and determine the extent of the injury in preoperative planning, rehabilitation and in the prevention of Knee Osteoarthritis. The authors have used relevant literature to support their findings and appropriate statistical analysis to evaluate the data. Overall, I find this article is suitable for indexing and adds new information to the fields of anatomy and surgery.

Is the work clearly and accurately presented and does it cite the current literature?

Yes

If applicable, is the statistical analysis and its interpretation appropriate?

Yes

Are all the source data underlying the results available to ensure full reproducibility?

Yes

Is the study design appropriate and is the work technically sound?

Yes

Are the conclusions drawn adequately supported by the results?

Yes

Are sufficient details of methods and analysis provided to allow replication by others?

Yes

Reviewer Expertise:

Clinical Anatomy

I confirm that I have read this submission and believe that I have an appropriate level of expertise to confirm that it is of an acceptable scientific standard.

# Response

**Published:** 2023-07-15

**Authors:** Vanishri Nayak

**Affiliations:** Kasturba Medical College, Manipal, India

We thank the reviewer for the expert comments. The morphometric data of this study are enlightening to the field of arthroscopic surgery. It has implications in analyzing the meniscal tears and discoid meniscus. The data can be used as morphological database for the Indian population.
